# The Ventilation of Hospitals

**Published:** 1887-04-30

**Authors:** Andrew Clark, Joseph Fayrer, Symes Thompson, Theodore Williams


### THE VENTILATION OF HOSPITALS.

BY SIR ANDREW CLARK, BART., M.D.; SIR JOSEPH FAYRER, K.C.S.I., M.D.; DR. SYMES THOMPSON; DR. THEODORE WILLIAMS; MR. BURDETT, AND OTHERS.

ON Wednesday evening, 20th April, the fifth general meeting of The Hospitals Association was held in the new building of the Hospital for Consumption, Brompton, when Dr. C. Theodore Williams, M.A., F.R.C.P., read the paper on "The Ventilation of Hospitals" published in this Journal last week. The chair was taken by Sir Andrew Clark, Bart., M.D., F.R.S., President of The Hospitals Association. There was a large attendance of members and friends, including Professor Arthur Gamgee, F.R.S., Sir Joseph Fayrer, K.C.S.I., M.D., Major-General Keatinge, C.S.I., V.C., Dr. Andrew Stephen, Dr. Symes Thompson (Senior Physician to the Brompton Hospital), Col. Montefiore, Mr. H. N. Custance (Sec., Hospital Sunday Fund), Mr. Pietro Michelli (Sec., St. Mary's, Paddington), Dr. E. Clifford Beale, Mr. Henry C. Burdett, Mr. J. P. Bartlett, Mr. C. L. Todd (Sec., St. George's), Mr. H. Dobbin (Sec., Brompton Hospital), Dr. Waugh (Medical Superintendent, Brompton Hospital), Mr. Thos. Redmayne and Mr. P. S. Webster of the London Hospital, Mr. J. F. C. Macready, F.R.C.S., Miss Franks (Matron, Kensington Infirmary), Mrs. Jenécké (Matron, Hospital for Incurables, Putney), Miss Lovett (Matron, Lock Hospital, Harrow Road), Miss Andrews and Miss Wood of the Lock Hospital, Mrs. G. D. Robinson (Suffolk General Hospital), Mrs. Peech, Miss E. Field, Miss Scott, Mr. J. B. Brunyate, Mr. Arthur B. Pierce, Dr., Mrs., and Miss Jelly, Mr. Michael Brophy, Miss Gwyn Jeffreys, Miss Jenkins, Mr. and Mrs. Westmacott, Miss Smith (Miller Memorial Hospital, Greenwich), Mr. and Mrs. John Lecky, Miss Milne, Miss M. Milne, Mr. J. Dacosta, Miss Bullen-Smith, and the Matron, the Clinical Assistants, and a large number of the Sisters and Nurses of the Brompton Hospital.

The CHAIRMAN, in introducing the lecturer, remarked that, in conversing with acquaintances upon the subject of hospital ventilation, he had been surprised to find it regarded as a matter of secondary moment. For his own part—and he had had some considerable experience of hospital life and work—he regarded it as a matter of cardinal importance for the well-being of hospitals and the success of their work. He supposed it was not too much to say that every hospital which opened its doors for the reception of patients had at least two duties to discharge to the public. First, it must not injure the patients whom it received, nor its officers and nurses; and secondly, it was its duty to do its best for the patients it received. He felt quite certain that, unless the ventilation of hospitals was most carefully attended to, serious injury would be inflicted, not only upon the patients, but also upon the officers and servants who were in the hospitals. It was not very long ago that in a great many hospitals every officer and servant might be seen with some form of blood poisoning; they had sore-throats, rheumatism, or some peculiar febrile movement, and it was the same with patients, sudden diseases arising in the hospitals, such as hospital gangrene, erysipelas, hospital sore-throat, and so forth. He knew himself of one hospital at the present time where one or other of the resident officers had some form of sore-throat, arising from impure air, so that he thought there could be no doubt that if our hospitals were not thoroughly ventilated, injury was inflicted upon patients, officers, and servants. It could not be doubted that, unless hospitals were thoroughly ventilated, the best could not be done for the patients. The first essential, if this was to be done, was that the patient should be placed under the best physiological conditions for resisting the encroachment of that with which he was threatened, and for repairing the damage which had been done. He was exceedingly glad that a distinguished member of The Hospitals Association had brought the subject before them that evening. He would not, therefore, stand any longer between the audience and Dr. Williams, whom he would call upon to read his paper. (Cheers.)

Dr. THEODORE WILLIAMS then read his paper on the "Ventilation of Hospitals."* During the reading of the paper, the lecturer invited Dr. Percy Franklin to explain certain results at which he had arrived in the examination, on different days and at different hours, of the air of the Laboratory at the Science School at South Kensington. The gas being turned down, the oxy-hydrogen light was thrown upon a screen at the back, and a series of plates, each measuring twelve square inches, were passed through the lantern. After detailing the process by which the organisms had been affixed to the slides, Dr. Franklin exhibited one plate which had been exposed for twenty-one minutes in the morning, while the students were in the laboratory, containing 228 moles, bacteria, etc. Another plate, exposed for the same length of time, but after the students had left, contained only 143. A third plate, exposed in the morning just after dusting, showed the large number of 428. A fourth plate, exposed some hours after the dust had subsided, contained 176, while a fifth plate, exposed in the evening after the students had all left, gave only ninety-five organisms. Some other plates demonstrated equally remarkable results as to the impurities existing in the air caused by the presence of individuals, the dusting of an apartment, etc. Dr. Williams subsequently supplemented his paper by giving the results of some observations which had been made at the Brompton Hospital by Dr. Marcet, F.R.S., on the amount of carbonic acid in the air of the galleries and wards of the new wing. On the occasion of his first visit (April 7), on testing the external air (the wind being strong east), he found the amount of $CO^2$ in 1,000 vols. to be .4, a state of purity which was extremely fair for London. He next tested the air of the east-end of the top gallery, with but few patients about, the result showing $CO^2$ .55 per 1,000 vols., while another experiment, near the centre, with thirty-three patients present, gave .65. In the centre of the Alexandra Gallery of this institution, with few patients about, the analysis revealed .467, and another one with a large number of patients, about .62. On April 18, Dr. Marcet found the external air (the wind being west, nearly calm) to contain carbonic acid .389 per 1,000 vols. In the Derby Ward, with one inlet for air, the atmosphere having suffered contamination by five patients for one hour and a half, the impurity rose to .795. In No. Twenty-five Ward, with two air inlets and five patients, the analysis showed .76, and another examination of the air of the west-end of the Alexandra Gallery gave .601.

At the conclusion of the paper, which appeared to arouse very considerable attention,

The CHAIRMAN remarked that he had listened with great interest and great advantage to the very interesting, able, and important paper. He made no doubt that they had all so listened, and therefore he was sure that they would justify him in conveying to Dr. Williams the most cordial vote of the meeting for the valuable communication which he had just laid before them. (Cheers.) The importance of the paper, it seemed to him, did not arise merely from the facts and conclusions which Dr. Williams had communicated, nor from the

* See THE HOSPITAL, April 23, p. 63.

further facts based on the results of the successful experiments carried out with so much skill. No small part was due to the fact that it had been spoken by a physician occupying a distinguished place in his art, one who was at once practically experienced and scientifically bred. This fact gave the paper an importance which otherwise it could never have had. Nevertheless, there was no subject of this kind on which nothing could be said " on the other side." They were honoured that evening by the presence of several distinguished men, especially by Sir Joseph Fayrer, whom he would ask to give them the benefit of his experience.

Sir JOSEPH FAYRER, who was very warmly received, confessed, on rising, that the last thing which he anticipated in coming to the meeting that night was that he should be called upon to make an address. He had come to listen to what he knew would be a most interesting paper, because, knowing the man, he judged that the thing must be worth coming down to Brompton to hear. However, in obedience to the order of the Chairman, he would endeavour to do what had been asked of him. He was quite delighted to be able to thoroughly emphasise all that their Chairman had said in reference to the importance of this subject, and to the value of the paper. He had listened to it with much interest, and had found that it grasped all the chief points of importance. He was not prepared to enter into details nor to give any figures, but he should be doing himself less than justice were he not to admit that considerable experience which he had gathered abroad and at home had taught him to take great interest in the subject they had been considering that evening, not only in reference to the ventilation of hospitals but of all public buildings and places inhabited by human beings. Bearing in mind the Association under whose auspices they had met, Dr. Williams had done well to select the ventilation of hospitals, in particular, but he could well have extended his observations to structures of other character. Many public buildings were still wanting to a great extent in common elements which we had a right to expect. He was quite satisfied that were these points attended to, the death-rate might be reduced ; its reduction was really at our own disposal. But he feared that, not until many other lecturers had given instruction and impressed upon their minds that which they ought to know, and engendered in them a belief in that which was only a matter of common sense, would people begin to understand that they could not live without pure air and cleanliness. His own experience of the subject—especially as regarded hospitals—had been gained in another country. What the lecturer had said applied also to tropical countries. In India (where he had gathered his experience) the hospitals were now being constructed in a manner much more adaptable to their requirements than was formerly the case. They were mostly simple rooms to contain from twenty-five to thirty people in each, with plenty of cubic space. The ideal was of course not reached. He believed himself that if every man, woman, and child could be separately placed, it would be better for them than in wards. In the great cities of India the hospitals were large. It was his fortune—perhaps he should say his misfortune—to be connected with one of the largest for many years. He did not reflect upon the authorities who constructed it,—they did the best according to their lights. As imitations of Greek temples and the like, they were all that could be desired, but they were totally unfitted for the purposes of hospitals, with their large wards opening into one another. Now, however, that great changes had taken place, the death-rate of the hospitals was consequently diminished. He was delighted to hear what had fallen from Dr. Williams with regard to the Brompton Hospital. He could never be satisfied with the construction of a hospital till he saw each ward distinct from the other. Such hospitals could be constructed, and our great military hospitals were being so built ; he might mention a typical case, a naval hospital at Malta. Of course it was needless for him to repeat that ventilation was absolutely essential. For your sick patient you must have a certain amount of cubic space. He must have, not 500 cubic feet to breathe from, but a great deal more ; 1,500 cubic feet was a proper amount : some hospitals had 2,000 cubit feet, and this was not a bit too much. The wards should also be cross-ventilated. They might depend upon it that the simpler the structure, the more successful the medical men would be. He was much obliged to Dr. Williams for having given him the opportunity of hearing a paper which was carefully put together and well summarised.

Dr. SYMES THOMPSON, in the name of the Committee of Management and the Medical Staff, assured the members of The Hospitals Association how very cordially they received them into their hospital that evening. It was an association which had already done, and was calculated to do, a vast deal of good, not only in this city, but throughout the world. He did feel, that in this matter of supplying pure air, they were dealing with one of the primary and most essential points for securing the success of a hospital. In meeting within the walls of that hospital, they had in an especial way an advantage which might not be found in other hospitals, inasmuch as in that institution the history of ventilation was very well taught. Dr. Thompson then referred at length to the controversy which once raged with respect to the proper mode of ventilation of the old building on the opposite side of the Fulham Road, and the part which his father played in that matter. He thought they had in the old building ample evidence of the diversity of views which the last half-century had seen. His father fought for the extraction system in opposition to what he might call the "pumping" system. These two systems worked for a time side by side. The only part of Dr. Williams's paper which he had to criticise, was where he said that the pumping system broke down and had to be replaced. He (Dr. Thompson) would rather say that the pumping system was never a success. It was far less satisfactory than the extracting system. There was in fact a radical difficulty in the pumping system In making their arrangements for the new hospital, they had found it important to draw the air from the open air in a number of places on all the floors, and it was now obtained in the wards direct from the outside and through the walls into the corridors, and distributed warmed, and not over-heated, to the building. When Dr. Williams said that the system of ventilation should be so arranged as to work successfully in summer as well as in winter, he mentioned a point which ought not to be neglected. All who had to do with hospitals were apt to get into a condition of routine. If we left these matters to subordinates—to engineers and officers who had no knowledge of the necessity for providing pure air for the patients, we should have evil results. They had at the Brompton Hospital a medical officer who was keenly alive to these matters. It was important also that they saw, not only that their apparatus was properly constructed, but that they kept it in order at all times and in all seasons. For example, at his hospital last Christmas the nurses decorated the wards, and in one instance the ventilator was so skilfully decorated that it ceased to be a ventilator at all. (Laughter.)

Sir JOSEPH FAYRER rose to supply an omission. He had intended to have corrected Dr. Williams with regard to his remark concerning the Indian punkah. The punkah did not ventilate by propulsion, but simply moved the air that was already there. What Dr. Williams doubtless meant was the thermantidote.

Mr. P. MICHELLI, the Secretary of St. Mary's Hospital, Paddington, said that he rather took an opposite view to that of Dr. Williams. He thought all hospitals should be built on the plan of cross-ventilation. In regard to the Tobin tube ventilator, he mentioned a case at his hospital where, on opening it, there were found a dead bird, a bone, and some dressings, over which the air had been passing. He was opposed to polished floors where the dirt was rubbed in.

Major-General KEATINGE referred to the system of sunk sills, where a board was put at the bottom of the window; the bottom sash could then be raised three or four inches, the window thus being converted into a Tobin tube without being opened. They had upwards of a hundred put in the new Nursing Home at the London Hospital. He wished to ask Dr. Williams to express his opinion on that mode of ventilation.

Mr. BURDETT was quite certain that all who had heard the paper had been impressed by its importance, in exact proportion to their knowledge of hospital sanitation. Of all the papers which had been read this session before the members of The Hospitals Association, probably that by Dr. Williams was the most practical and the most useful, and he personally felt that all who were interested in hospitals were greatly indebted to him for the way in which he had raised this question. He himself had had some experience of the working of artificial and natural ventilation in hospitals of all kinds in this and other countries. With reference to the question of impurities in hospitals, within a few yards of where they were then met there was a hospital—the Cancer—where, years ago, it was thought that if you had encaustic tiles placed upon the walls you had all that could be desired. As a matter of experience it was found that encaustic tiles were no preventive to impurities, and hospitalism prevailed to such an extent that it was decided to remove the tiles. That was a most important experience. Mr. Burdett proceeded to mention another case, at the Northampton Hospital, where "economic" principles prevailed to such an extent that it was decided to place new floors upon the old ones. At the Bristol Infirmary also the "economists" said it was not worth while to remove the old floors and put new floors down, but that it would be adequate to place new floors upon the old. He would warn the people of Bristol that, if they decided to adopt the arguments of the so-called "economists," and save a few pounds, they could not hope in the surgical wards to be free from gangrene and erysipelas. The paper they had just heard read referred to Sir James Simpson's statistics on hospitalism, and to those of Holmes and Erichsen, but there were later statistics, namely, those of cottage hospitals. Sir James Simpson was right in his belief of the advantage of segregating the sick in relatively small wards. Speaking of the German surgeons, Mr. Burdett observed that they had proved that the chief aid to successful treatment was cleanliness. He remembered years ago going over the Victoria Hospital with the late Dr. Peacock, who at first advocated the artificial heating of wards. He told him himself that, as the result of long experience, he was forced to the conclusion that artificial heating and ventilation were not beneficial to patients, but the reverse. He had himself visited hospitals on the Continent and in America, where the climate was so varied as to render artificial ventilation in the wards absolutely necessary. Many in the room would agree with him that we had no greater authority than Dr. Billings on hygiene. Now, Dr. Billings had written a book on ventilation, and he had embodied in that book all the experiments which had been made; he had, in fact, carried out more experiments than any other living man. When he (Mr. Burdett) was in America, Dr. Billings desired that he should go with him to inspect a hospital which had been built under his own supervision a little way outside Washington. The fan system of ventilation was in force at this hospital. When Dr. Billings and he arrived at the hospital, they found that the wards were stuffy. The cause soon became clear. On examination they found that the engine was out of repair, and cobwebs were on the fan. The medical superintendent had, in fact, not used it, and the result was that, in spite of the expenditure incurred and the thoroughness of the system, when it passed into the hands of an ordinary administrator it became a failure, because he did not believe in it. He was therefore opposed to artificial means of ventilation, if they could be avoided.

Dr. WILLIAMS, in replying, said he was very glad to hear that there was an honourable opposition, because he should be very sorry that all he laid down should be carried *nemine contradicente*. He was quite aware that there was a difference of opinion on some points. He claimed, he thought, their chairman on his side, and so, too, to a large extent, Sir Joseph Fayrer. He saw that he was very strongly in favour of cross-ventilation. In the Brompton Hospital they had many difficulties to contend against in the matter of ventilation. They had to make the wards rather small. Large wards did not answer in a consumption hospital, as there was a great deal of coughing, which might, in large wards, keep other patients awake. They had no wards above eight beds, and some had only one, two, or three. Dr. Symes Thompson had given a most interesting account of the various stages by which the ventilation of the hospital had been experimented upon. One point to be observed about the hospital was that they always had an artificial system of ventilation. He could not say anything as regarded Mr. Keith Young's objections, because he had not seen them, but, as the views of an eminent architect, they were doubtless worthy of consideration. Mr. Michelli was in favour of natural ventilation. On this point he thought they were not quite agreed upon terms. Mr. Michelli spoke of the ventilation by means of fireplaces; that he (Dr. Williams) certainly regarded as artifical ventilation. Mr. Michelli had referred to the dead bird found in the ventilator. Of course no system of ventilation would do unless it was properly looked after, any more than a chimney would work without being seen to. As to the polished floors, he confessed that he had not heard any actual argument against them. The system of ventilation which Major-General Keatinge had referred to was what he had described in his paper as Hinckes Bird's method of raising the window. It did not, however, allow of a large amount of air coming in between the two sashes. Mr. Burdett's remarks were always valuable. He had said a great deal that supported his paper, especially his reference to the encaustic tiles at the Cancer Hospital. What we wanted in hot weather was to create a current of air; that was done at the Brompton Hospital by keeping the shafts up to a great heat (80 or 90 degs.). A current was created all the year round. In winter you could do very well if you kept up your fire without the regulation system of extraction by the shaft. In summer that method broke down. In countries where the windows could be open all the year round, it was a different thing. In the Brompton Hospital they could not possibly do without artificial ventilation, because they were obliged to keep up a temperature of 60 to 62 degs. through the winter.

The CHAIRMAN said he would just make two short observations. The first was, that it had become plain from the paper and the various speakers, that cleanliness and ventilation were not only essential to the success of a hospital and the safety of those who dwelt therein, but that it was absolutely criminal to disregard them. The second was that of the two systems of ventilation—the natural and the artificial—it was sometimes not possible to have the natural. Either in respect to the character of the building, or in respect to the season of the year, natural ventilation might not be effectual, and therefore they had to have recourse to the artificial. He was connected for many years with the London Hospital, where he had four wards under his charge. Once on a time a great stranger came to visit the hospital, and was taken round by the Committee. When he came to one of his wards, he observed, "You are a little close," and expressed himself as shocked that the windows were not open, adding that that was the proper mode of ventilation. One of the Committee ventured to suggest that the patients might take a cold, to which the great stranger retorted, that "no one took a cold from ventilation." So the windows were opened, and five days afterwards he (Sir Andrew) found nearly half the patients suffering from acute inflammation, and three of them died.

